# Meta-analysis and systematic review of coronary vasospasm in ANOCA patients: Prevalence, clinical features and prognosis

**DOI:** 10.3389/fcvm.2023.1129159

**Published:** 2023-03-13

**Authors:** Janneke Woudstra, Caitlin E. M. Vink, Diantha J. M. Schipaanboord, Etto C. Eringa, Hester M. den Ruijter, Rutger G. T. Feenstra, Coen K. M. Boerhout, Marcel A. M. Beijk, Guus A. de Waard, Peter Ong, Andreas Seitz, Udo Sechtem, Jan J. Piek, Tim P. van de Hoef, Yolande Appelman

**Affiliations:** ^1^ Department of Cardiology, Amsterdam Cardiovascular Sciences, Amsterdam UMC Location Vrije Universiteit Amsterdam, Amsterdam, Netherlands; ^2^ Laboratory of Experimental Cardiology, University Medical Center Utrecht, Utrecht University, Utrecht, Netherlands; ^3^ Department of Physiology, Amsterdam Cardiovascular Sciences, Amsterdam UMC Location Vrije Universiteit Amsterdam, Amsterdam, Netherlands; ^4^ Department of Physiology, Maastricht University, Cardiovascular Research Institute Maastricht, Maastricht, Netherlands; ^5^ Department of Clinical and Experimental Cardiology, Amsterdam Cardiovascular Sciences, Amsterdam UMC Location University of Amsterdam, Amsterdam, Netherlands; ^6^ Department of Cardiology, Robert Bosch Hospital, Stuttgart, Germany

**Keywords:** coronary vasospasm, ANOCA, acetylcholine provocation test, sex, microcirculation

## Abstract

**Background:**

Coronary artery spasm (CAS), encompassing epicardial and microvascular spasm, is increasingly recognized as cause of angina in patients with non-obstructive coronary artery disease (ANOCA). However, various spasm provocation testing protocols and diagnostic criteria are used, making diagnosis and characterization of these patients difficult and interpretation of study results cumbersome. This review provides a structured overview of the prevalence, characterization and prognosis of CAS worldwide in men and women.

**Methods:**

A systematic review identifying studies describing ANOCA patients with CAS was performed. Multiple outcomes (prevalence, clinical features, and prognosis) were assessed. Data, except for prognosis were pooled and analysed using random effects meta-analysis models.

**Results:**

Twenty-five publications (*N* = 14.554) were included (58.2 years; 44.2% women). Percentages of epicardial constriction to define epicardial spasm ranged from >50% to >90%. Epicardial spasm was prevalent in 43% (range 16–73%), with a higher prevalence in Asian vs. Western World population (52% vs. 33%, *p* = 0.014). Microvascular spasm was prevalent in 25% (range 7–39%). Men were more likely to have epicardial spasm (61%), women were more likely to have microvascular spasm (64%). Recurrent angina is frequently reported during follow-up ranging from 10 to 53%.

**Conclusion:**

CAS is highly prevalent in ANOCA patients, where men more often have epicardial spasm, women more often have microvascular spasm. A higher prevalence of epicardial spasm is demonstrated in the Asian population compared to the Western World. The prevalence of CAS is high, emphasizing the use of unambiguous study protocols and diagnostic criteria and highlights the importance of routine evaluation of CAS in men and women with ANOCA.

**Systematic Review Registration:**

https://www.crd.york.ac.uk/prospero/display_record.php?RecordID=272100.

## Introduction

Coronary artery spasm (CAS), encompassing epicardial and microvascular spasm, is increasingly recognized as the substrate for both chronic and acute coronary syndromes especially in patients with angina and no obstructive coronary artery disease (ANOCA). Importantly CAS is associated with repeat hospitalizations, impaired quality of life and higher incidence of adverse cardiac events (1) To date, the established method for the diagnosis of CAS is invasive spasm provocation testing using intracoronary administration of acetylcholine (Ach) or ergonovine (Ergo) (2) and has recently been endorsed in the European guidelines for evaluation of chronic coronary syndromes in the absence of obstructive coronary artery disease (3). However, a consistent spasm provocation testing protocol for detecting epicardial and microvascular spasm is still lacking, leading to various protocols used across continents, and even between expert centres. This involves differences in both the applied doses, administration protocols, including the diagnostic agent used (4). Moreover, diagnostic criteria to define a pathological response to spasm provocation testing have long remained ambiguous. Taken together, different testing protocols and diagnostic criteria hamper comparison of study results, including assessment of ethnic and sex differences in CAS. To unify the diagnosis of CAS, the *Coronary Vasomotion Disorders International Study Group* (COVADIS) proposed diagnostic criteria in 2015, which since, unfortunately, have not been widely implemented in both clinical practice and research initiatives (5, 6). As a result, extrapolation of study results remains cumbersome. Considering the variety of data available in contemporary literature, we want to provide clinicians with a state-of-the art overview of the data currently available. Therefore, the aim of the present report is to provide a structured overview of the available evidence regarding the prevalence, clinical characteristics, and prognosis of CAS in men and women with ANOCA across different continents.

## Methods

### Protocol and registration

This systematic review was conducted in accordance with the Preferred Reporting Items for Systematic Reviews and Meta-Analysis (PRISMA) guidelines (7). The study protocol was prospectively developed and registered at The International Prospective Register of Systematic Reviews (PROSPERO, crd.york.ac.uk/PROSPERO), registration number CRD42021272100).

### Search strategy

Search strategies were developed in consultancy with a medical librarian and PubMed, Embase.com and Web of Science were searched. Thesaurus terms and free-text words, including synonyms and closely related words, were used for the following concepts: “coronary vasospasm”, “ANOCA” and “INOCA”. In Supplementary Appendix 1. of the supplementary data the search strategy is shown. The search was conducted on May 20, 2021 and updated in February 2022. To remove duplicates, records were imported into Endnote X9.1 (Clarivate Analytics, Philadelphia, PA).

### Study selection

Studies had to fulfil the following criteria: (1) evaluate patients with evidence of epicardial or microvascular spasm; (2) evaluate ANOCA patients, to create more homogeneity in the included study populations, we only included patients with chronic coronary syndromes with no obstructive coronary artery disease and excluded patients with ST-elevation myocardial infarction and non-ST-elevation myocardial infarction, since this is a different patient group and may influence prevalence and prognosis of CAS; (3) adults ≥18 year; (4) describe a study population of ≥45 patients in order to limit the inclusion of too small studies; (5) English literature; (6) completed trials; (7) published after 1980, to limit inclusion of overaged studies. Conference abstracts and case reports were excluded. All identified literature was initially screened on title and abstract and additionally the full text by two reviewers independently (JW and CV) using Rayyan. Any disagreements were discussed, and final decision was reached by consensus with a third author (YA). Also, a forward and backward citation chasing was performed for other potentially relevant studies on all papers included in this review. In case multiple publications of the same study population were included, the publication containing most complete data was included. Other publications concerning the same study population were used to retrieve missing data. Study quality was evaluated by using the Joanna Briggs Institute critical appraisal tools (Supplementary file, Supplementary Tables S1-S4).

### Data extraction and outcome measure

The following data was extracted from the selected studies, when available: (1) publication details: study author, recruitment period, year of publication, journal reference; (2) study design and timing of data collection (prospective/retrospective); (3) study population: country of publication; (4) participant characteristics: sample size, age, sex, clinical features; (5) details regarding CAS; (6) details regarding the spasm provocation test (7) outcome measures. A detailed description of the data extraction and outcome measures can be found in Supplementary Appendix 2. of the supplementary files.

### Subgroup and statistical analysis

Included studies were structured and analysed according to the different outcome measures, separate between men and women with ANOCA, and in the different continents when sufficient data was available. Data for prevalence and clinical features were pooled and analysed using random effects meta-analysis models. Meta-analysis models were considered feasible when ≥3 studies described similar outcomes. Heterogeneity in the studies was assessed using I2 statistics. For studies including both ANOCA patients with and without CAS, studies reporting clinical features in microvascular and epicardial spasm and studies including data regarding epicardial spasm in women and men, the summary odds ratios (OR) or mean difference and 95% confidence interval were calculated, since three or more studies described these outcome measurements. Due to heterogeneity, meta-analysis was not feasible for the prognostic data. R Statistics (version 4.0.3) was used for analyses.

## Results

### Search results

**Figure 1:**
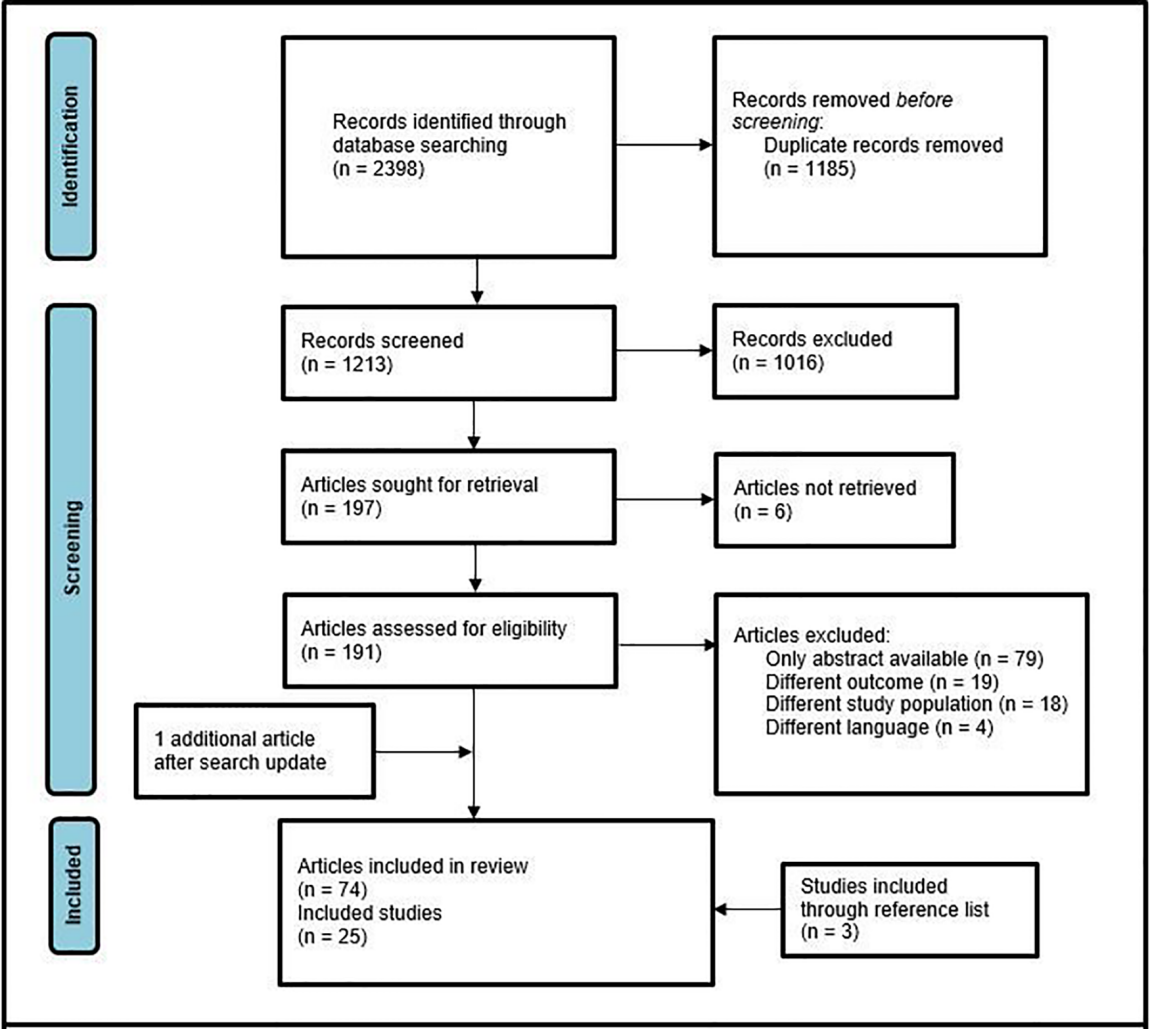
PRISMA flow diagram of study selection.

Through initial search 2,398 records were identified. After removal of duplicates, 1,213 potentially relevant citations were screened on title and abstract. Hereafter, full text evaluation of 191 articles was performed, identifying 74 original. After exclusion of studies with overlapping study populations, 21 articles remained. Three additional articles were included after forward and backward citation chasing. One more recently published study was included after the search update in February 2022, resulting in 25 included studies (Figure 1) (8–32). The selected studies are summarized in Table 1.

**Table 1a.** Included articles regarding epicardial spasm from Western World populations

Author (year)	Study design	Recruitment period	Total cohort (*n* = 4287)	ANOCA patients (*n* = 3890)	Mean age (years) ±SD	Female *n* (%)	Control group	Diagnostic agent	Diagnosis spasm	Dosages
Country (Trial)										
**EUROPE**
Arrebola-Moreno (2014)	Cohort	2012	50	50	60.5 ± 8.9	31 (62)	NA	Ach	>75% spasm, symptoms & ischemic ECG	2,20,100,200 ug 3 min ic into LAD
* Spain* (*8*)										
Aziz (2017) (9)	Cohort	2007–2014	1379	1379	61.9 ± 11.1	806 (58.4)	AP	Ach	>75% spasm, symptoms & ischemic ECG	2,20,100,200 ug 3 min ic into LAD
* Germany*										
Bory (1996) (26)	Cohort	1977–1991	277	277	53.6 ± 9.3	71 (25.6)	NA	Ergo	>50% spasm, without additional criteria	1,2,3,6 ug/kg-1 injection
* France*										
Castello (1988) (10)	Case-Control	NR	112	35	51.3	106 (94.6)	oCAD	Ergo	>75% spasm, without additional criteria	0.1, 0.2, 0.3 mg bolus into ascending aorta
* Spain*										
Coma-Canella (2005)	Cohort	NR	162	162	54 ± 11	53 (32.7)	NA	Ergo	>50% spasm, without additional criteria	1,5,10,30 ug 1 min ic into RCA, LAD
* Spain* (12)										
Figueras (2013) (13)	Cohort	1980–2010	657	657	55.5 ± 10.3	284 (43.2)	AP	Ergo & Ach	>50% spasm, without additional criteria and/or transient ST segment changes during pain	Ergo: 0,05 mg up to 0.85 mg iv; Ach 20,50,100 ug ic
* Spain*										
Ford (2019) (27) –	RCT	2016–2017	391	151	60.9 ± 10.0	111 (73.5)	AP	Ach	>90% spasm, symptoms & ischemic ECG	100 ug bolus ic into LAD, 50 ug bolus ic into RCA
* Scotland (CORMICA)*										
Fournier (1989) (28)	Cohort	1984–1988	108	108	46 ± 9	43 (39.9)	NA	Ergo	>75% spasm, without additional criteria	50,100, 200 ug 3 min iv and 25 ug ic
* Spain*										
Jansen (2021) (14)	Cohort	2019–2021	264	264	58 ± 8	228 (86.4)	NA	Ach	>90% spasm, symptoms & ischemic ECG	2,20,100,200 ug 1–3 min ic into LAD
* Netherlands*										
Schoenenberger (2016)	Cohort	1997–2008	718	718	56.4	357 (49.7)	NA	Ach	>50% spasm, without additional criteria	NR
* Swiss* (*20*)										
Scholl (1986) (21)	Case-Control	NR	120	40	50 ± 9	27 (22.5)	oCAD, HC	Ergo	>75% spasm, symptoms & ischemic ECG	0.04, 0.16, 0.2 mg
* France*										
**AUSTRALIA**
Sheikh (2018) (22)	Cohort	2013–2016	49	49	53.9 ± 11.0	38 (78)	NA	Ach	>90% spasm, symptoms & ischemic ECG	25,50,100 ug bolus 20 sec ic into LM
* Australia*										

Ach, acetylcholine; ANOCA, angina with no obstructive coronary arteries; Ergo, ergonovine; ECG, electrocardiogram; ic, intra-coronary; iv, intravenous; LAD, left anterior descending artery; LM, left main; RCA right coronary artery; n, number; NA, not applicable; NR, not reported; oCAD, obstructive coronary artery disease; RCT, randomized controlled trial; SD, standard deviation; ug, microgram.

**Table 1b.** Included articles regarding epicardial spasm from Asian populations.

Author (year)	Study design	Recruitment period	Total cohort (*n* = 11688)	ANOCA patients (*n* = 10664)	Mean age (years) ±SD or IQR	Female n (%)	Control group	Diagnostic agent	Diagnosis spasm	Dosages
Country (Trial)										
Choi (2019) (11)	Cohort	2004–2014	5890	5890	55.3 ± 12.4	3187 (54.1)	NA	Ach	>70% spasm, without additional criteria	2,20,100,200 ug 1 min ic into LAD
* Korea*										
Lee (2017) (15)	Cohort	2003–2014	986	986	56 (IQR 46–63)	148 (15.0)	NA	Ergo	>90% spasm, symptoms or ischemic ECG	20,40,80 mg ic into LAD
* Korea*										
Mitsugi (2007) (17)	Case-Control	NR	67	67	61.6 ± 9.2	40 (59.7)	AP	Ach	>90% spasm, without additional criteria	10,30,100 ug 30s ic into LAD
* Japan*										
Mohri (1998) (17)	Cohort	1994–1997	117	117	63 (IQR 54–68)	59 (50.4)	NA	Ach	>75% spasm, without additional criteria	10,30,100 ug 30s ic into LAD
* Japan*										
Nishimiya (2021) (29)	Cohort	2013–2018	329	329	61.4	153 (46.5)	NA	Ach	>90% spasm, symptoms or ischemic ECG	20,50,100 ug 20s ic into LAD
* Japan*										
Nishio (2017) (18)	Cohort	2012–2014	65	65	65.5	28 (43.1)	HC	Ach	>90% spasm, symptoms or ischemic ECG	20,50,100 ug 20s ic into LAD
* Japan*										
Odaka (2017) (19)	Cohort	2011–2014	198	198	60.2 ± 13.3	82 (41.4)	NA	Ach	>90% spasm & ischemic ECG	20,50,100 ug 20s ic into LAD
* Japan*										
Sato (2013) (30)	Cohort	1991–2010	1877	1877	63.0 ± 11.0	776 (47.4)	NA	Ach	>75% spasm & ischemic ECG	20,50,100 ug 20s ic into LAD
* Japan*										
Suda (2019) (23)	Cohort	2014–2017	187	187	63.2 ± 12.3	74 (39.5)	NA	Ach	>90% spasm, symptoms & ischemic ECG	20,50,100 ug 20s ic into LAD
* Japan*										
Sueda (2015) (31)	Cohort	1991–2012	1440	416	64.4 ± 10.8	193 (46.3)	NA	Ach	>99% spasm, without additional criteria	20,50,100 ug 20s ic into LAD
* Japan*										
Sugiishi (1993) (32)	Cohort	1975–1990	351	351	55.5	76 (21.7)	NA	Ergo	>75% spasm, without additional criteria	0.1, 0.2, mg iv
* Japan*										
Sun (2005) (24)	Cohort	1995–2000	131	131	59.8	69 (52.7)	NA	Ach	>75% spasm, without additional criteria	10,30,100 ug 30s ic into LAD
* Japan*										
Yamanaga (2014) (26)	Case-Control	2011–2014	50	50	61.3	24 (48.0)	NA	Ach	>90% spasm, symptoms & ischemic ECG	20,50,100 ug 30s ic into LAD
* Japan*										

Ach, acetylcholine; ANOCA, angina with no obstructive coronary arteries; Ergo, ergonovine; ECG, electrocardiogram; ic, intra-coronary; iv, intravenous; IQR, inter quartile range; LAD, left anterior descending artery; RCA right coronary artery; n, number; NA, not applicable; NR, not reported; RCT, randomized controlled trial; SD, standard deviation.

### Patient characteristics

The 25 included studies describing patients with CAS had a combined total study population of 15.975 patients, encompassing 14.554 ANOCA patients, since three studies compared patients with obstructive coronary artery disease with ANOCA patients. The total study population had a mean age of 58.2 years of which 44.2% were women (*n* = 7.064). Twelve studies (*n* = 4.287) described Western World populations (mean age 57.7; 40.3% women) (8–10, 12–14, 20–22, 26–28) and 13 studies (*n* = 11.688) described Asian populations (mean age 58.4; 42.0% women) (11, 15–19, 23, 25, 29–32).

### Prevalence of CAS

In the included studies, different definitions were used for the diagnosis of epicardial and microvascular spasm (Table 1). Concerning epicardial spasm, in 5 of the included studies the diagnostic criteria were in accordance with the COVADIS criteria. Twenty studies applied less strict criteria, i.e., vasoconstriction of <90% to define epicardial spasm or criteria were used that did not include concomitant ischemic ECG changes or recognizable symptoms during provocation testing. For microvascular spasm similarly different definitions were used, 7 of 10 studies used diagnostic criteria in accordance with the COVADIS criteria. A detailed description of the different diagnostic criteria used can be found in Supplementary Appendix 3 of the supplementary files.

**Figure 2:**
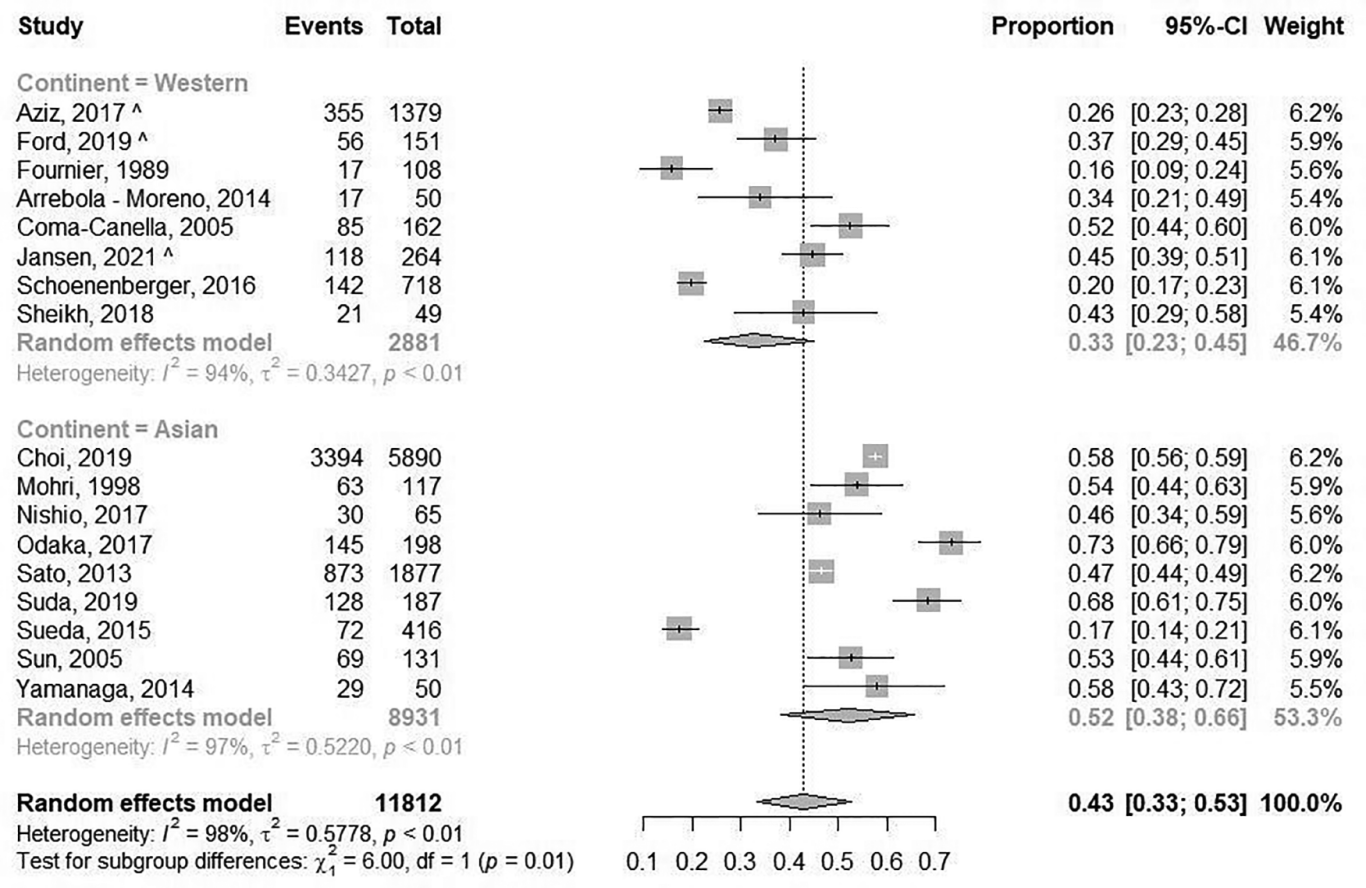
Prevalence of epicardial spasm. Forest plot of published studies examining the prevalence of epicardial spasm using random effects meta-analysis divided by continent of publication. Data presented as percentage (%) and 95% confidence intervals (CI; %).

**Figure 3:**
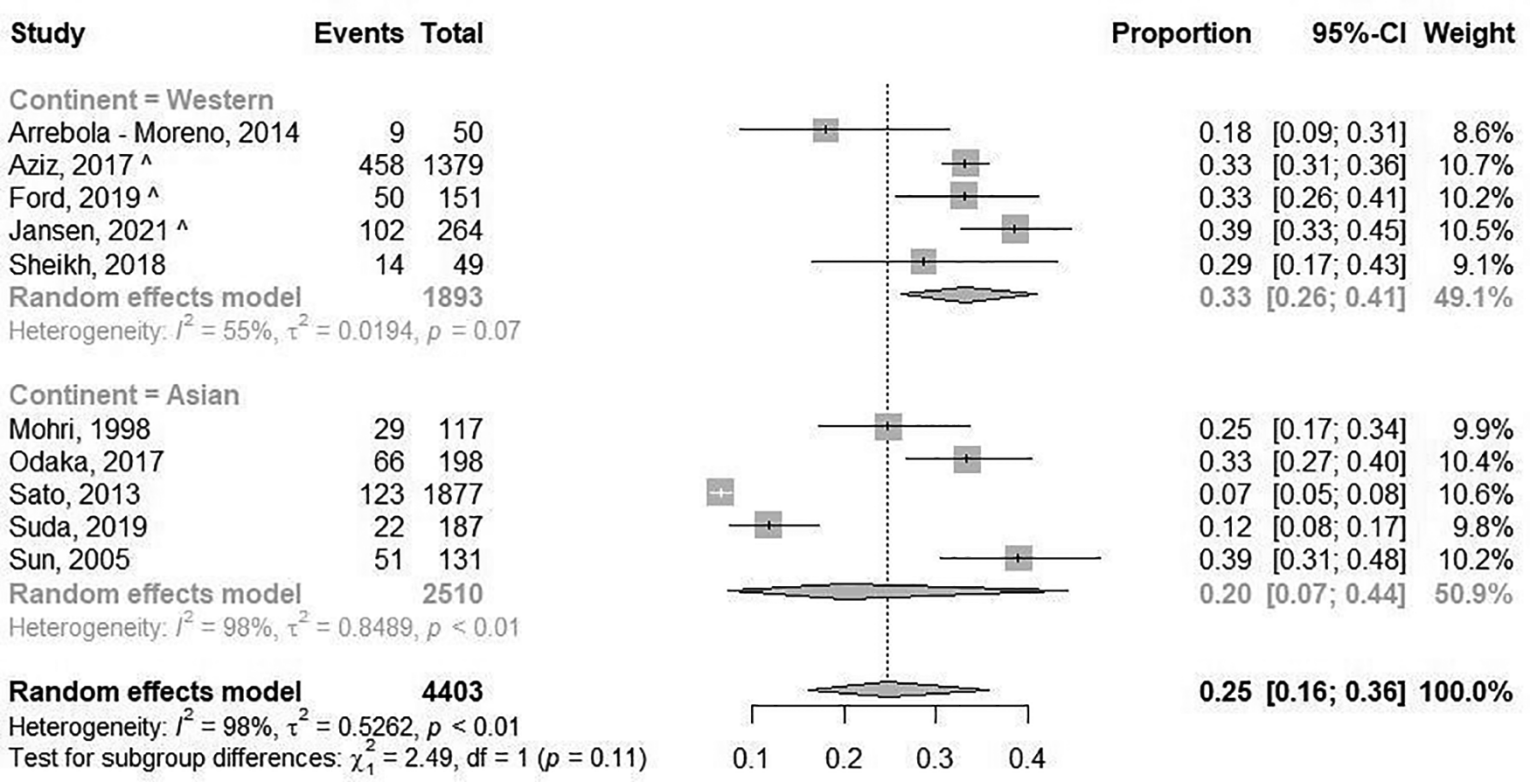
Prevalence of microvascular spasm. Forest plot of published studies examining the prevalence of microvascular spasm using random effects meta-analysis. Data presented as percentage (%) and 95% confidence intervals (CI; %).

The prevalence of epicardial spasm in ANOCA patients was reported in 17 studies, including 11.762 ANOCA patients with a mean age of 58.1 years of which 52.2% were women (Supplementary Table S5) (8, 9, 11, 12, 14, 17–20, 22–25, 27, 28, 30, 31). Studies reported an incidence of epicardial spasm between 16%–73% and an overall prevalence of epicardial spasm (*n* = 5.614) of 43% [95% confidence interval (CI), 33%–53%], based on random effects analysis (Figure 2). When studies only using Ach as diagnostic agent and using similar diagnostic criteria (i.e., 75% or 90% epicardial vasoconstriction with ischemic ECG changes and symptoms) were included in a separate random effects analysis, studies reported an overall prevalence of 44% (95% CI, 30%-58%) (Supplementary Figure S1) (8, 9, 14, 22, 23, 25, 27). In addition, Supplementary Figure S2 shows the prevalence of epicardial spasm in studies published the past five years, reporting a prevalence of 50% (95% CI, 36%-64%). Furthermore, a separate analysis including studies within the funnel-plot (Supplementary Figure S3) was performed, reporting a prevalence of 42% (95% CI, 36%-48%) (Supplementary Figure S5). The prevalence of microvascular spasm was reported in 10 studies (*n* = 4,403 ANOCA patients) with a mean age of 61.9 years of which 51.6% were women(8, 9, 14, 17, 19, 22–24, 27, 30). Based on random effects analysis, studies reported an overall prevalence of 25% (95% CI, 16%–36%) (Figure 3). A separate analysis including studies within the funnel plot (Supplementary Figure S4) reported a prevalence of 24% (95% CI 15%-37%) (Supplementary Figure S6).

**Central Illustration:**
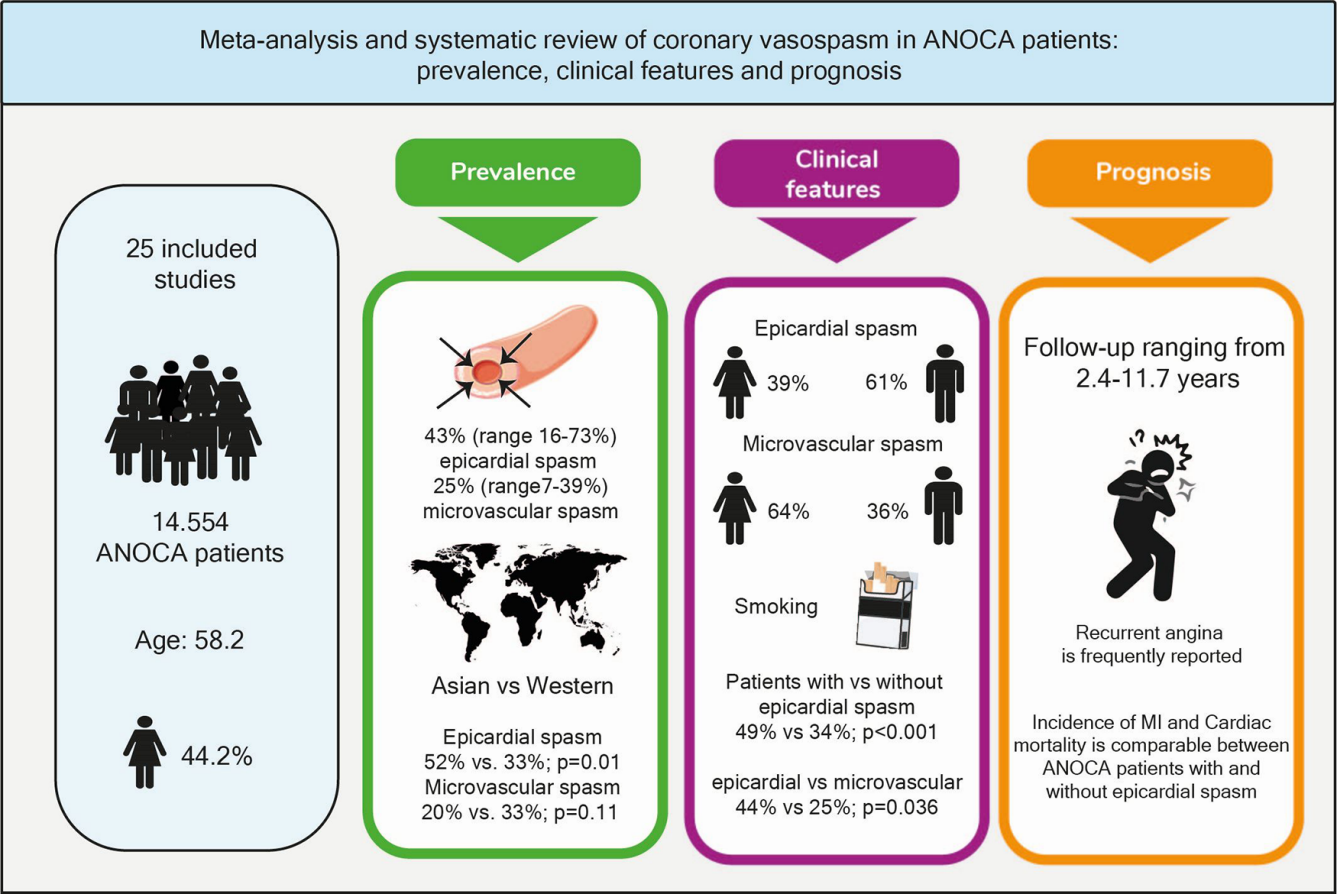
ANOCA, angina with no obstructive coronary arteries; CAS, coronary artery spasm; MI, myocardial infarction.

### CAS in different continents

When studies were evaluated based on ethnicity, random effects analysis showed a significant different prevalence of epicardial spasm between Western World populations of 33% (8 studies, *n* = 2,881 ANOCA patients, 95% CI, 23%–45%) (8, 9, 12, 14, 20, 22, 27, 28), vs. 52% (95% CI, 36%–66%) and Asian populations(9 studies, *n* = 8,931 ANOCA patients)(11, 17–19, 23–25, 30, 31), (*p* = 0.014) (Figure 2). When microvascular spasm was compared between continents, random effects analysis showed a prevalence in Western World populations of 33% (5 studies, *n* = 1,893 ANOCA patients, 95% CI, 26%–41%) (8, 9, 14, 22, 27), vs. 20% (95% CI, 16%–36%) in Asian populations (5 studies, *n* = 304 ANOCA patients) (17, 19, 23, 24, 30), which did not reach statistical significance (Figure 3). A numerical difference that did not reach statistical significance was seen in the prevalence of epicardial spasm in women from Western World populations compared to women from Asian populations (57% vs. 35%, respectively) (Table 2). Additionally, diabetes mellitus was seen significantly more often in Asian populations with epicardial spasm (21% vs. 13%, *p* < 0.01) and dyslipidaemia in Western World populations with epicardial spasm (56% vs. 40%, *p* < 0.01) (Table 2). Due to insufficient data it was not feasible to compare CV risk factors in microvascular spasm patients between Western World and Asian publications.

**Table 2.** Cardiovascular risk factors in ANOCA patients with epicardial spasm in Western World and Asian populations.

	Western World populations	Asian Populations	Total	*P* value
Number of patients	949	2771	2729	
Age (mean years, 95% CI)	57 (54–65)	62.4 (59–64)	61 (59–63)	0.29
Women (%, 95% CI)	57 (29–82)	35 (24–47)	42 (31–55)	0.08
Hypertension (%, 95% CI)	50 (37–64)	45 (39–51)	47 (42–53)	0.37
Dyslipidaemia (%, 95% CI)	56 (42–70)	40 (27–55)	47 (37–57)	<0.01
DM (%, 95% CI)	13 (7–24)	21 (17–26)	17 (14–22)	<0.01
Smoking (%, 95% CI)	49 (31–67)	50 (35–65)	49 (39–60)	0.89

Seventeen studies examining cardiovascular risk factors from Western and Asian populations patients were included in this random effects meta-analysis. ANOCA, angina with no obstructive coronary arteries; DM, diabetes mellitus.

### CAS and sex differences

Sixteen studies compared ANOCA patients with and without epicardial spasm (16 studies; *n* = 6117). Of the ANOCA patients with epicardial spasm 39% (95% CI 29%- 50%) were women, compared to 53% (95% CI, 47%-59%) of ANOCA patients without epicardial spasm [OR 0.56 (CI 0.37–0.84); *P* < 0.01] (Table 3)(8, 9, 13, 16–20, 23–25, 27, 29–32). Four studies compared ANOCA patients with and without microvascular spasm (*n* = 466) (8, 9, 17, 24). In microvascular spasm patients 64% (95% CI, 27%-90%) were women compared to 48% (95% CI, 20%-77%) of ANOCA patients without microvascular spasm, which did not reach statistical significance (Supplementary Table S6). In addition, 3 articles (*n* = 2437) compared CV risk factors between female and male epicardial spasm patients, which is shown in Supplementary Table S8 (9, 15, 31). Due to insufficient data it was not feasible to compare CV risk factors between female and male microvascular spasm patients.

**Table 3.** Clinical features in ANOCA patients with and without epicardial spasm.

	ANOCA patients with ES (*n* = 2721) %(95% CI)	ANOCA patients without ES (*n* = 3396) %(95% CI)	Mean difference/ OR (95% CI) & P Value
Age (mean years)	61 (59 – 63)	60 (58 – 61)	1.14 (−0.18, 2.45), *p* = 0.086
Women (%)	39% (29%–50%)	53% (47%–59%)	0.56 (0.37,0.84), *p* = 0.008
Hypertension (%)	53% (47%–59%)	49% (42%–56%)	0.90 (0.76, 1.07), *p* = 0.214
Dyslipidaemia (%)	48% (38%–58%)	42% (32%–53%)	1.23 (0.96,1.57), *p* = 0.090
DM (%)	18% (14% –23%)	15% (13%–18%)	1.11 (0.94,1.3), *p* = 0.211
Smoking (%)	49% (38%–60%)	34% (28%–41%)	2.01 (1.40, 2.88), *p* < 0.001

Seventeen studies examining cardiovascular risk factors between ANOCA patients with and without epicardial spasm were included in this random effects meta-analysis. ANOCA, angina with no obstructive coronary arteries; CI, confidence interval; DM, diabetes mellitus; ES, epicardial spasm; OR, odds-ratio.

### Clinical characteristics of ANOCA patients with CAS

Seventeen studies (*n* = 6.117) reported CV risk factors in patients with (*n* = 2.721) and without epicardial spasm (*n* = 3.396) (Table 3) (8, 9, 12, 13, 16–20, 23–25, 27, 29–32). In the epicardial spasm group (49%; 95% CI, 38%–60%) smoking occurred significantly more often compared to patients without epicardial spasm (34%; 95% CI, 28%-41%) [OR 2.01 (1.40–2.88), *p* < 0.01] (Table 3). Other CV risk factors (i.e., hypertension, dyslipidaemia, diabetes mellitus) were similar between the two groups. Four studies reported clinical features for epicardial and microvascular spasm patients, which can be found in Supplementary Table S7 (8, 9, 17, 24).

### Prognosis of epicardial CAS

Eleven studies reported long-term outcomes of CAS patients, describing 12.058 patients (mean age 57.4; 47.4% women). Due to heterogeneity combining study results was not feasible, however an overview is presented in Table 4. Eight studies described the incidence of cardiac death (*n* = 5.968), with a follow-up ranging from 2.4 to 11.7 years. Three studies analysed the occurrence of cardiac death in ANOCA patients with and without epicardial spasm, with a follow-up period of 7.2, 11.3 and 11.7 years (1, 13, 20). None of the aforementioned found a significant difference in the occurrence of cardiac death between both groups. Eight studies (*n* = 4.737) reported the incidence of myocardial infarction (MI) in patients with epicardial spasm, with a follow-up ranging from 1 to 11.7 years. Three studies analysed the occurrence of MI between ANOCA patients with and without epicardial spasm, with a follow-up period of 7.2, 11.3 and 11.7 years. Figueras et al. and Schoenenberger et al. did not report a significant difference between both groups, while Seitz et al. reported that MI occurred more often in epicardial spasm patients compared to patients with microvascular spasm and to ANOCA patients without CAS (1, 13, 20). MACE was reported in 5 studies (*n* = 3.512), with a follow-up ranging from 1 to 4.4 years. Three studies defined MACE as cardiac death, nonfatal MI and unstable angina, the other two used the same definition as the aforementioned, but added hospitalization for heart failure or stroke and transient ischemic attacks. MACE was reported in 268 patients (12%) with epicardial spasm, more than half of all MACE was caused by hospitalization for unstable angina ranging from 52% to 90% of MACE. In addition, Nishimiya et al. and Sato et al. reported a better prognosis in patients with diffuse spasm compared to focal spasm (29, 30). Recurrent angina was reported in various ways and the key findings are summarized in Table 4. However, overall, recurrent angina occurred frequently during follow up, ranging from 10%–53%. The prognosis of microvascular spasm was rarely reported, however Seitz et al., reported that microvascular spasm is an independent predictor of recurrent angina after a median follow-up period of 7.2 years (1). A causal relationship between medical treatment and prognosis was not reported in the included studies.

**Table 4.** Overview of studies reporting prognosis.

** **	Cardiac Mortality			MACE			Myocardial infarction			Mean follow-up (years)	Recurrent angina
Study	Total ANOCA Cohort	ANOCA with ES	ANOCA without ES	Total Cohort	ANOCA with ES	ANOCA without ES	Total Cohort	ANOCA with ES	ANOCA without ES		(Summarized findings)
*Total cohort (n)*	*n* (%)	*n* (%)	*n* (%)	*n* (%)	*n* (%)	*n* (%)	*n* (%)	*n* (%)	*n* (%)		
Aziz (2017)^a^	3 (0.5)	2 (0.8)	1 (0.3)	NR	NR	NR	7 (1.3)	7 (2.8))	0 (0)	7.2	53% of ES and 57% of MS patients had angina during follow up. Recurrent angina occurred in 41% of ANOCA patients without CAS.
* (n = 847)*										(6.5 – 7.9)	
Bory (1996)	10 (3.6)	10 (3.6)	NA	NR	NR	NR	18 (6.5)	18 (6.5)	NA	7.4	21% of ES patients had persistent angina at follow up requiring repeated CAG.
* (n = 277)*										(1– 16.5)^c^	
Choi (2019)	NR	NR	NR	NR	NR	NR	NR	NR	NR	3.4	The risk of sustained angina pectoris was associated with coronary artery spasm and was 9.6% in patients with ES.
* (n = 5890)*											
Figueras (2013)	25 (3.8)	15 (5.5)	10 (2.6)	NR	NR	NR	24 (3.7)	9 (3.3)	15 (3.9)	11.7	The occurrence of angina at follow-up was 46% in ES patients and in ANOCA patients without ES 61%.
* (n = 657)*										(6.1– 17)^b^	
Ford (2019)^a^	NR	NR	NR	17 (11.3)	17 (11.3)	NA	4 (2.6)	4 (2.6)	NA	1.0	Stratified medical therapy in ANOCA patients leads to significant improved QoL and angina (measured with SAQ) improvement after ICFT.
* (n = 151)*											
Lee (2017)	13 (1.3)	13 (1.3)	NA	191 (19.4)	191 (19.4)	NA	16 (1.9)	16 (1.9)	NA	4.4	17% of ES patients had rehospitalisation for repeated angina at follow up, similar between men and women.
* (n = 986)*										(2.1–7.1)	
Nishimiya (2021)	0 (0)	0 (0)	0 (0)	17 (5.2)	17 (6.9)	0 (0)	3 (1.0)	3 (1.3)	0 (0)	2.9	Hospitalization for unstable angina or HF occurred in 6% of ES patients and none of control patients.
* (n = 329)*										(2.5–3.1)	
Sato (2013)	NR	3 (0.3)	NR	NR	43 (5.9)	NR	NR	6 (0.7)	NR	4.1 ± 1.6	4% of ES had unstable angina during follow up.
* (n = 1877)*											
Schoenenberger (2016)	4 (0.6)	4 (2.8)	0 (0)	NR	NR	NR	5 (1.2)	3 (2.1)	2 (0.7)	11.3 ± 2.7	23% of CAS patients had unchanged or worsened angina during follow up.
* (n = 718)*											
Sheikh (2018)	NR	NR	NR	NR	NR	NR	NR	NR	NR	1/12	No relationship appeared between epicardial spasm and recurrent angina in ANOCA patients.
*(n = 49)*											
Suda (2019)	1 (0.5)	NR	NR	10 (5.4)	NR	NR	NR	NR	NR	2.4	In the overall ANOCA population MACE occurred in 5% at follow up and consisted for 90% of hospitalization for unstable angina.
*(n = 187)*										(1.7–3.1)	
Total	56 (1.5)	47 (1.6)	11 (0.9)	235 (14.4)	268 (12.0)	0 (0)	77 (2.4)	66 (2.2)	17 (1.6)		
Total comparative studies	32 (1.4)	21 (2.3)	11 (0.9)	17 (5.4)	17 (6.9)	0 (0)	39 (2.0)	22 (2.4)	17 (1.6)		

Follow-up data are presented in mean ± SD or median (interquartile range).

^a^ Data retrieved from other studies from the same study cohort

^b^ incidence reported at first 11.7 years.

^c^ range instead of IQR; ANOCA, angina with no obstructive coronary arteries; CAG, coronary angiography; CAS, coronary artery spasm; ES, epicardial spasm; HF, heart failure; ICFT, invasive coronary function test; IMR, index of microcirculatory resistance; MACE, major adverse cardiac events; MI, myocardial infarction; MS, microvascular spasm; NR, Not reported; SAQ, seattle angina questionnaire; QoL, quality of life.

## Discussion

This systematic review provides an overview of the prevalence, clinical features and prognosis of CAS in ANOCA patients worldwide. Our data demonstrate that: (1) The prevalence of epicardial spasm in ANOCA patients is 43% (ranging from 16%–73%) and 25% for microvascular spasm (ranging from 7%–39%); (2) A significantly higher prevalence of epicardial spasm is reported in Asian populations compared to Western World populations (52% vs. 33%) while a comparable prevalence is reported for microvascular spasm (20% vs. 33%); (3) While men were more likely to have epicardial spasm (61%), women were more likely to have microvascular spasm (64%); (4) The incidence of cardiac mortality and MI was low and comparable in ANOCA patients with and without epicardial spasm, MACE was dominated by recurrent angina (Central illustration).

### Prevalence of CAS and differences in protocols and definitions

A wide variation in prevalence was seen in our review, which could be related to differences in used protocols and inclusion criteria as demonstrated in Table 1.

First, the wide variety of spasm provocation test protocols could have led to differences in prevalence. It is well known that the occurrence of CAS is dependent on Ach concentrations, which increase with higher dosages of Ach and higher injection speed (32).

Second, numerous CAS definitions were applied, which influence the diagnosis and thus prevalence of CAS. For example, Sueda et al. in 2015 used very strict diagnostic criteria, i.e., spasm was defined as coronary vasoconstriction of more than 99%, resulting in a low prevalence of 17% (31). However, we found that, in studies using similar diagnostic criteria the prevalence of epicardial spasm was comparable with the prevalence using all the included studies (Supplementary Figure S2 and S2). To overcome this problem, the COVADIS working group published diagnostic criteria for epicardial and microvascular spasm in 2015 (5). As numerous articles were published before this publication it can partly explain the differences we found in definitions and protocols. However, since the COVADIS criteria are the strictest criteria used worldwide, they are not always applied in daily practice, possibly because clinicians are divided how to treat the large group of patients only partly meeting the diagnostic criteria (34), while studies using milder criteria still report high incidences of recurrent angina and MACE (1, 11). Nonetheless, the risk of implementing too lenient criteria is over diagnosis.

Third, differences in patient selection could have led to the variety of prevalence. Unfortunately, additional information regarding patient selection, for example the duration and nature of angina symptoms, was rarely reported. In line with our findings, a recently published review by Mileva et al. reported a similar but slightly lower prevalence of spasm of 40% and of microvascular spasm of 24% (35). This difference could be explained by the fact that MINOCA patients were also included in the review of Mileva et al., suggesting a slightly higher prevalence of CAS in ANOCA patients compared to MINOCA patients. In addition to the study of Mileva et al., our study includes additional information as we performed a subgroup analysis of studies using similar Ach testing protocols and diagnostic criteria. Also, this review provides an overview of clinical features and prognosis and investigated differences between continents and sexes.

In addition to the above mentioned explanations for the differences in prevalence, it is also important to realize that in the majority of studies only epicardial spasm was reported, while our review reported a prevalence of microvascular spasm of 25%. It is reasonable to assume that the prevalence of microvascular spasm is underestimated since the coexistence of epicardial and microvascular spasm is difficult to diagnose. When after Ach provocation and additional nitroglycerin an Ach re-challenge is performed microvascular spasm can be unmasked. This is not daily practice and also was not performed in the included studies in this review (4).

### CAS and ethnicity

Our review observed differences in prevalence of epicardial spasm between Asian and Western World population. Previously multiple hypothesis for this observed difference in prevalence have been described. First, environmental and genetic factors may play a pivotal role. For example, it has been proposed in literature that the coronary arteries of Japanese CAS patients have an increased coronary artery basal tone compared to Caucasian patients, that may be related to an increased hyperreactivity in reaction to provocation test stimuli (33). Second, underestimation of the prevalence of CAS in Western World countries is suggested due to the fact that spasm provocation testing is performed more routinely only since recently (34). In the *2019 ESC Guidelines for the diagnosis and management of chronic coronary syndromes* the spasm provocation test has been recommended as a diagnostic tool in ANOCA patients, also in the recently published EAPCI expert consensus document concerning invasive coronary function testing (3, 36). Third, in Asian countries an injection speed of 20–30 s is most commonly used, which is higher compared to Western World countries and as described previously the occurrence of CAS increases with higher injection speed (4, 32). Forth, in line with our analysis, an increased incidence of diabetes mellitus in Asian populations is frequently described and it has been suggested that diabetes mellitus plays a role in the development of CAS due to the fact that it is associated with endothelial dysfunction and subsequent CAS (36).

### CAS and sex differences

While the majority of ANOCA patients are women, we found that epicardial spam occurred more often in men. Previous research using intracoronary ultrasound has shown that the presence of subtle atherosclerotic infiltration is associated with CAS in ANOCA (37). In men, coronary atherosclerosis is more prevalent at a younger age compared to women and might explain the higher prevalence of epicardial spasm in men (38). Smoking, which is more common in men is a well-known risk factor for CAS, due to inactivation of nitric oxide (32). Furthermore, it is known that, in women cardiovascular risk, including epicardial spasm, increases after menopause (39). Possibly a part of the included women is still protected by their premenopausal state. Unfortunately information regarding menopausal state was lacking. In line with our analysis, previous studies also reported a greater burden of coronary microvascular dysfunction, including microvascular spasm, among women (14). The underlying cause is not defined yet, but lower myocardial mass, smaller body size and more tortuous coronary arteries with thinner walls are suggested to play a role (40).

### Prognosis

The prevalence of cardiac mortality and MI seems comparable low between ANOCA patients with and without epicardial spasm. However, both groups have an impaired prognosis compared to the normal population, most likely due to the fact that a part of the ANOCA patients without epicardial spasm have coronary microvascular dysfunction or microvascular spasm, which is not investigated in all studies. This is supported by Jespersen et al. describing an increased risk of all-cause mortality and MACE in ANOCA patients compared to a population without ischemic heart disease (41). In addition the most important finding is that patients experience a high number of recurrent angina during follow-up, which is in line with previous studies such as the CorMicA-trial. This trial was the first controlled clinical trial that randomized ANOCA patients based on invasive coronary function testing to stratified treatment or to standard care (27). After 6 months patients in the stratified treatment group reported a significant improvement in angina and quality of life (27). The frequently reported recurrent angina in the included studies of our review, might be due to insufficient medical treatment in CAS patients. Three studies reported the use of calcium-channel blockers during follow-up, in which a high variability was seen in the reported use. In two of these studies, less than half of all patients used calcium-channel blockers at follow-up. The reason for this low use of calcium-channel blockers is not reported, however raises two concerns. First, the effect of calcium-channel blockers might not be sufficient in CAS and new treatment options are necessary. Second, these patients are not prescribed to adequate tailored treatment including sometimes much higher doses than usually given in patients with obstructive CAD.

## Limitations

Several limitations of our study should be acknowledged. First, an important limitation is the heterogeneity of the diagnostic criteria and spasm provocation test protocols used, which most likely let to the heterogeneity between studies in the meta-analysis. Also, publication bias could not be excluded (Supplementary Figures S3, S4). However, an additional analysis, including studies within the funnel-plot, showed a similar prevalence as compared to the prevalence using all the included studies (Supplementary Figure S5, S6). Second, the prevalence depends on the patient population selected for the spasm provocation testing. Included studies in this review most often used a selected population of patients. Third, the prognostic data should be interpreted cautious in view of the different follow-up periods and limited insight in medical treatment. Fourth, most included studies compared epicardial spasm in ANOCA patients with ANOCA patients without epicardial spasm. This could have led to distorting evidence considering different diagnostic criteria were used in the included studies. Patients categorized as suffering from epicardial spasm could have been diagnosed as ANOCA without epicardial spasm in studies using stricter criteria. Fifth, limited data were available regarding duration and type of angina complaints of the included ANOCA patients.

## Conclusion

This systematic review provides an overview of ANOCA patients with epicardial and microvascular spasm based on the available data to date and demonstrates a high prevalence of both entities. Men are more likely to have epicardial spasm, while women are more likely to have microvascular spasm and a lower prevalence of epicardial spasm was found in Western World population compared to Asian population. Disabling symptoms during life are the most important issue for ANOCA patients while further having a rather good prognosis. As the prevalence of CAS is high in ANOCA patients additional coronary spasm provocation testing should follow when obstructive coronary artery disease is ruled out in both men and women. In this, distinguishing between epicardial and microvascular spasm will lead to correct diagnosis and tailored treatment. Furthermore, this study emphasizes the need for unambiguous diagnostic criteria and protocols in ANOCA patients and the implementation of those.

## Data Availability

The original contributions presented in the study are included in the article/**Supplementary Material**, further inquiries can be directed to the corresponding author/s.
