# Supplementary material for: Meta-analysis and systematic review of coronary vasospasm in ANOCA patients: Prevalence, clinical features and prognosis
Source: Front Cardiovasc Med. 2023 Mar 13;10:1129159. doi: 10.3389/fcvm.2023.1129159 (PMC10041338; doi:10.3389/fcvm.2023.1129159)
Supplement: Supplementary file 1 [file Datasheet1.docx]

***Supplementary file***

**Meta-analysis and systematic review of coronary vasospasm in ANOCA patients: prevalence, clinical features and prognosis**

**Janneke Woudstra^1^, MD; Caitlin E.M. Vink^1^, MD; Diantha J.M. Schipaanboord^2^, Msc; Etto C. Eringa^3,4^; Hester M. den Ruijter^2^, PhD; Rutger G.T. Feenstra^5^, MD; Coen K.M. Boerhout^5^, MD; Marcel A.M. Beijk^5^, MD, PhD; Guus A. de Waard^1^, MD, PhD; Peter Ong, MD^6^; Andreas Seitz, MD^6^; Udo Sechtem, MD^6^; Jan J. Piek^5^, MD, PhD; Tim P. van de Hoef ^2^, MD, PhD; Yolande Appelman^1*^, MD, PhD.**

**Correspondence**

Y. Appelman, MD, PhD

Email: [y.appelman@amsterdamumc.nl](mailto:y.appelman@amsterdamumc.nl)

**Index:**

1. **Supplementary Data**

Appendix 1: Search strategies

Appendix 2: Supplementary methods

Appendix 3: Supplementary results

1. **Supplementary Figures and Tables**

Appendix 4:

- Table S1. Quality assessment using JBI’s critical appraisal tool for cross sectional studies
- Table S2. Quality assessment using JBI’s critical appraisal tool for case control studies
- Table S3. Quality assessment using JBI’s critical appraisal tool for cohort studies
- Table S4. Quality assessment using JBI’s critical appraisal tool for randomized controlled trials

Appendix 5:

- Table S5. Included articles regarding spasm prevalence.
- Table S6. Cardiovascular risk factors in ANOCA patients with and without microvascular spasm
- Table S7. Cardiovascular risk factors in epicardial and microvascular spasm patients
- Table S8. Cardiovascular risk factors in female and male ANOCA patients with epicardial spasm

Appendix 6:

- Figure S1: Prevalence of epicardial spasm when studies used Acetylcholine as diagnostic agent and used the following diagnostic criteria; 1) epicardial vasoconstriction of 75% or 90%, 2) ischemic ECG changes, 3) symptoms.
- Figure S2: Prevalence of epicardial spasm when only studies from 2017 or later are included in the analysis.
- Figure S3: Publication bias: funnel plot epicardial spasm
- Figure S4: Publication bias: funnel plot microvascular spasm
- Figure S5: Prevalence of epicardial spasm when only studies within the funnel plot are included in the analysis.
- Figure S6: Prevalence of microvascular spasm when only studies within the funnel plot are included in the analysis.

1. **Supplementary Data**

**Appendix 1. Search strategies**

PubMed Medline search May 20, 2021.

Yield: 781 titles

[Mesh] = Medical subject headings

[tiab] = words in title or abstract or author keywords

| #1 | ("Angina Pectoris, Variant"[Mesh] OR "Coronary Vasospasm"[Mesh] OR "vasospastic angina" [tiab] OR "Prinzmetal angina*" [tiab] OR "princemetal angina*"[tiab] OR "Coronary Vasospasm*"[tiab] OR "Coronary artery spasm*"[tiab] OR "Coronary arterial spasm*"[tiab] OR "epicardial spasm*"[tiab] OR "microvascular spasm*"[tiab] OR "Intracoronary acetylcholin*"[tiab] OR "acetylcholine provocat*"[tiab] OR "spasm provocation test*"[tiab] OR "Coronary spasm*"[tiab] OR "coronary artery vasoconstrict*"[tiab] OR "coronary arterial vasoconstrict*"[tiab] OR "coronary constrict*"[tiab] OR "VSA"[tiab] OR "Variant angina"[tiab]) | 9301 |
| --- | --- | --- |
| #2 | ("nonobstruct*"[tiab] OR "non obstruct*" [tiab] OR "non-obstruct*" [tiab] OR "no obstruct*"[tiab] OR "no-obstruct*"[tiab] OR "unobstruct*" [tiab] OR "un obstruct*" [tiab] OR "unobstruct*" [tiab] OR "un obstruct*" [tiab] OR "nonocclusive" [tiab] OR "non occlusive"[tiab] OR "X syndrome"[tiab] OR "syndrome X"[tiab] OR "CSX"[tiab] OR "INOCA"[tiab] OR "ANOCA"[tiab] OR "NOCAD"[tiab] OR “normal coronary arter*” [tiab]) | 23548 |
| #3 | #1 AND #2 | 781 |

Embase.com search May 20, 2021.

Yield: 1211 titles.

/exp = EMtree keyword with explosion

ti,ab,kw = words in title or abstract or author keywords

NEXT/n = Requests terms that are within n words of each other in the order specified

| #1 | 'coronary artery spasm'/exp OR 'variant angina pectoris'/exp OR 'coronary artery constriction'/exp OR 'vasospastic angina':ti,ab,kw OR 'prinzmetal angina*':ti,ab,kw OR 'princemetal angina*':ti,ab,kw OR 'coronary vasospasm*':ti,ab,kw OR 'coronary artery spasm*':ti,ab,kw OR 'coronary arterial spasm*':ti,ab,kw OR 'epicardial spasm*':ti,ab,kw OR 'microvascular spasm*':ti,ab,kw OR 'intracoronary acetylcholin*':ti,ab,kw OR 'acetylcholine provocat*':ti,ab,kw OR 'spasm provocation test*':ti,ab,kw OR 'coronary artery vasoconstrict*':ti,ab,kw OR 'coronary arterial vasoconstrict*':ti,ab,kw OR 'coronary constrict*':ti,ab,kw OR ‘VSA’:ti,ab,kw OR ‘Variant angina’:ti,ab,kw | 14045 |
| --- | --- | --- |
| #2 | 'nonobstruct*':ti,ab,kw OR 'non obstruct*':ti,ab,kw OR 'non-obstruct*':ti,ab,kw OR 'no obstruct*':ti,ab,kw OR 'unobstruct*':ti,ab,kw OR 'un obstruct*':ti,ab,kw OR 'non occlusive':ti,ab,kw OR ‘X syndrome’:ti,ab,kw] OR ‘syndrome X’:ti,ab,kw OR ‘CSX’:ti,ab,kw OR ‘INOCA’:ti,ab,kw OR ‘ANOCA’:ti,ab,kw OR ‘NOCAD’:ti,ab,kw OR ‘normal coronary arter*’:ti,ab,kw | 34451 |
| #3 | #1 and #2 | 1211 |

Web of Science search May 20, 2021.

Yield: 406 titles

TS= Topic Search, words in title, abstract, author keywords or ‘keywords plus’

| #1 | TS=(“vasospastic angina” OR “Prinzmetal angina*” OR “princemetal angina*” OR “Coronary Vasospasm*” OR “Coronary artery spasm*” OR “Coronary arterial spasm*” OR “epicardial spasm*” OR “microvascular spasm*” OR “Intracoronary acetylcholin*” OR “acetylcholine provocat*” OR “spasm provocation test*” OR “coronary artery vasoconstrict*”OR “coronary arterial vasoconstrict*” OR “coronary constrict*”) | 7348 |
| --- | --- | --- |
| #2 | TS= (“nonobstruct*” OR “non obstruct*” OR “non-obstruct*” OR “no obstruct*” OR “unobstruct*” OR “un obstruct*” OR “unobstruct*” OR “un obstruct*” OR ”non occlusive” OR “non occlusive” OR "X syndrome" OR "syndrome X" OR "CSX" OR "INOCA" OR "ANOCA" OR "NOCAD" OR “normal coronary arter*” ) | 26806 |
| #3 | #1 and #2 | 406 |

**Appendix 2. Supplementary methods**

Data extraction and outcome measure

The following data was extracted from the selected studies, when available: (1) publication details: study author, recruitment period, year of publication, journal reference; (2) study design and timing of data collection (prospective/retrospective); (3) study population: country of publication; (4) participant characteristics: sample size, age, sex, clinical features (defined as cardiovascular risk factors with the definition used in each included study), comorbidities; (5) details regarding CAS; epicardial or microvascular spasm (defined according to the definition used in each included study), cardiac symptoms; (6) details regarding the spasm provocation test: diagnostic agent used, dosages diagnostic agent, time of infusion, site of infusion, administration route (i.e. intra-venous or intracoronary), COVADIS criteria used (i.e. reproduction of symptoms and ischemic ECG changes for microvascular spasm and epicardial spasm is defined as the aforementioned accompanied by >90% epicardial vasoconstriction); (7) outcome measures: a. prevalence of epicardial and microvascular spasm, compared between Asian and Western World study populations; b. clinical features including age, sex, traditional cardiovascular risk factors compared between sex and Asian and Western World countries, and; c. prognosis, encompassing angina symptoms, cardiac mortality, myocardial infarction, major adverse cardiovascular events (MACE), defined as the definition used in each included study, time to event. In addition, when studies described CAS patients with obstructive and non-obstructive coronary artery disease only ANOCA patients were included in the analyses.

**Appendix 3. Supplementary results**

In the 25 included studies describing CAS, different definitions were used for the diagnosis of epicardial (Tables 1 and 2). The percentage of epicardial constriction for the definition of epicardial spasm ranged from >50% to >90%. In 5 of the included studies the diagnostic criteria were in accordance with the COVADIS criteria. In 20 studies the criteria were less strict, i.e. vasoconstriction of less than 90% to define epicardial spasm or diagnostic criteria used did not include concomitant ischemic ECG changes or recognizable symptoms. In 12 studies, only vasoconstriction was necessary for diagnosis of epicardial spasm, meaning that symptoms and ischemic ECG changes were not included in the diagnostic criteria. In 13 studies a combination of vasoconstriction with symptoms and/or ischemic ECG changes was used for the diagnosis. For microvascular spasm similarly different definitions were used across the included studies. Seven studies defined microvascular spasm as angina symptoms and ischemic ECG changes during the provocation test, without epicardial vasoconstriction which was defined either less than 75% or 90% constriction. Three studies included myocardial lactate production in the diagnosis of microvascular spasm, either as sole criterion or in combination with symptoms and/or ECG changes. Besides differences in diagnostic criteria, also differences in testing protocols were seen: (1) Eighteen studies used Ach and 8 studies used Ergo as diagnostic agent; (2) In four studies the highest dose of Ach was 200 µg into the left coronary artery. In the other 19 studies, a lower maximal dose was used to diagnose epicardial spasm, most commonly 100 µg of Ach into the left coronary artery; (3) The administration route was primarily intracoronary, except for 5 studies that used an intravenous administration route (Ergo only); (4) The infusion time ranged from 20 seconds to 3 minutes when an intracoronary bolus injection was administered.

**2. Supplementary figures and Tables**

**Appendix 4. Quality assessment using JBI’s critical appraisal tool**

| **Cross-sectional** | **Inclusion criteria clear** | **Subjects and setting described** | **Valid**  **Exposure measured** | **Objective measurement** | **Confounding factors**  **(2)** | **Outcome measures** | **Appropriate statistical analysis** | **Score** |
| --- | --- | --- | --- | --- | --- | --- | --- | --- |
| Arrebola-Moreno et al. | Yes | Yes | Yes | Yes | No (2) | Yes | Yes | 6/8 |
| Aziz et al. | Yes | Yes | Yes | Yes | NA | Yes | Yes | 6/6 |
| Bory et al. | No | Yes | Yes | No | No (2) | Yes | Yes | 4/8 |
| Coma-Canella et al. | Yes | Yes | Yes | Yes | No (2) | Yes | Yes | 6/8 |
| Fournier et al. | Yes | Yes | Yes | Yes | NA | Yes | Yes | 6/6 |
| Konst et al. | Yes | Yes | Yes | Yes | No (2) | Yes | Yes | 6/8 |
| Mohri et al. | Yes | Yes | Yes | Yes | No (2) | Yes | Yes | 6/8 |
| Montone et al. | Yes | Yes | Yes | Yes | No (2) | Yes | Yes | 6/8 |
| Odoka et al. | Yes | Yes | Yes | Yes | Yes(2) | Yes | Yes | 8/8 |
| Sueda et al. | Yes | Yes | Yes | Yes | No(2) | Yes | Yes | 6/8 |
| Sun et al. | Unclear | Yes | Yes | Yes | Yes(2) | Yes | Yes | 5/8 |

**Table S1. Quality assessment using JBI’s critical appraisal tool for cross-sectional studies**

**Table S2. Quality assessment using JBI’s critical appraisal tool for case control studies**

| **Article** | **Groups comparable** | **Matched appropriate** | **Similar inclusion criteria** | **Exposure measurement**  **(2)** | **Confounding factors**  **(2)** | **Outcome assessment** | **Exposure period** | **Appropriate statistical analysis** | **score** |
| --- | --- | --- | --- | --- | --- | --- | --- | --- | --- |
| Castello et al. | No | No | Yes | Yes (2) | No (2) | Yes | Yes | Yes | 6/10 |
| Figueras et al. | Yes | No | Yes | Yes/No | Yes/No | Yes | Yes | Yes | 7/10 |
| Mitsugi et al. | Yes | No | Yes | Yes (2) | No (2) | Yes | Yes | Yes | 7/10 |
| Nishio et al. | Yes | Yes | Yes | Yes (2) | Yes (2) | Yes | Yes | Yes | 10/10 |
| Scholl et al. | Yes | Yes | Yes | Yes (2) | No (2) | Yes | Yes | Yes | 8/10 |
| Sugiishi et al. | Unclear | No | Yes | Yes (2) | Unclear/Yes | Yes | Yes | Yes | 7/10 |
| Yamanaga et al. | Yes | No | Yes | Yes (2) | Yes (2) | Yes | Yes | Yes | 9/10 |

**Table S3. Quality assessment using JBI’s critical appraisal tool for cohort studies**

| **Article** | **Groups comparable** | **Exposure measurement**  **(2)** | **Confounding factors**  **(2)** | **Free of outcome** | **Outcome assessment** | **Follow-up (3)** | **Appropriate statistical analysis** | **score** |
| --- | --- | --- | --- | --- | --- | --- | --- | --- |
| Bory et al. | NA | Yes/No | No | Yes | Yes | No/Yes/Yes | Yes | 6/10 |
| Choi et al. | Yes | Yes (2) | Yes/Unclear | Yes | Yes | Yes/no/No | Yes | 8/11 |
| Lee et al. | Yes | Yes (2) | No (2) | Yes | Yes | Yes/No/No | Yes | 7/11 |
| Nishimiya et al. | Yes | Yes (2) | Unclear (2) | Yes | Yes | Yes/No/No | Yes | 7/11 |
| Sato et al. | Yes | Yes/Unclear | Yes (2) | Yes | Yes | Yes/No/No | Yes | 8/11 |
| Schoenenberger et al. | Yes | Yes/Unclear | Yes (2) | Yes | Yes | Yes/Yes/No | Yes | 9/11 |
| Sheikh et al | Yes | Yes (2) | No (2) | NA | Yes | No/Yes/NA | Yes | 6/9 |
| Suda et al. | Yes | Yes (2) | Yes (2) | Yes | Yes | Yes/No/No | Yes | 9/11 |

| **Table S4. Quality assessment using JBI’s critical appraisal tool for randomized controlled trials** | | | | | | | | | | | |
| --- | --- | --- | --- | --- | --- | --- | --- | --- | --- | --- | --- |
|  | **Randomization** | **Treatment concealed (3)** | **Treatment groups similar** | **Outcome assessors blinded** | **Groups identical** | **Follow up** | **Intention to treat** | **Outcome measures (2)** | **Appropriate statistical analysis** | **Appropriate design** | **Score** |
| Ford et al. | Yes | No | Yes | Yes | Yes | Yes | Yes | Yes | Yes | Yes | 10/13 |

**Appendix 5.**

**Table S5. Included articles regarding spasm prevalence.**

| **Author (year)**  **Country (Trial)** | **ANOCA (n=11812)** | **Epicardial spasm**  **(n=5624)**  **n (%)** | **Microvascular spasm**  **(n=924)**  **n (%)** | **Mean age (years) ±SD or IQR** | **Female**  **ANOCA**  **n (%)** | **Diagnostic agent** | **Diagnosis epicardial spasm** | **Diagnosis microvascular spasm** |
| --- | --- | --- | --- | --- | --- | --- | --- | --- |
| **EUROPE** | | | | | | | | |
| Arrebola-Moreno (2014) | 50 | 17 (34) | 9 (18) | 60.5±8.9 | 31 (62) | Ach | >75% spasm, symptoms & ischemic ECG | Symptoms & ischemic ECG |
| Aziz (2017) | 1379 | 355 (26) | 458 (33) | 61.9±11.1 | 806 (58.4) | Ach | >75% spasm, symptoms & ischemic ECG | Symptoms & ischemic ECG |
| Coma-Canella (2005) | 162 | 85 (52.2) | NR | 54±11 | 53 (32.7) | Ergo | >50% spasm, without additional criteria | NA |
| Ford (2019) | 151 | 56 (37) | 50 (33) | 60.9±10.0 | 111 (73.5) | Ach | >90% spasm, symptoms & ischemic ECG | Symptoms & ischemic ECG |
| Fournier (1989) | 108 | 17 (16) | NR | 46±9 | 43 (39.9) | Ergo | >75% spasm, without additional criteria | NA |
| Jansen (2021) | 264 | 118 (44.7) | 102 (38.6) | 58±8 | 228 (86.4) | Ach | >90% spasm, symptoms & ischemic ECG | Symptoms & ischemic ECG |
| Schoenenberger (2016) | 718 | 142 (20.1) | NR | 56.4 | 357 (49.7) | Ach | >50% spasm, without additional criteria | NA |
| **AUSTRALIA** | | | | | | | | |
| Sheikh (2018) | 49 | 21(43) | 14(29) | 53.9±11.0 | 38 (78) | Ach | >90% spasm, symptoms & ischemic ECG | Symptoms & ischemic ECG |
| **ASIA** | | | | | | | | |
| Choi (2019) | 5890 | 3394 (57.6) | NR | 55.3±12.4 | 3187 (54.1) | Ach | >70% spasm, without additional criteria | NA |
| Mohri (1998) | 117 | 63 (54) | 29 (25) | 63 (IQR 54-68) | 59 (50.4) | Ach | >75% spasm, without additional criteria | Symptoms or ischemic ECG |
| Nishio (2017) | 65 | 30 (65) | NR | 65.5 | 28 (43.1) | Ach | >90% spasm, symptoms or ischemic ECG | NA |
| Odaka (2017) | 198 | 145 (73) | 66 (33) | 60.2± 13.3 | 82 (41.4) | Ach | >90% spasm & ischemic ECG | Myocardial lactate production |
| Sato (2013) | 1877 | 873 (50) | 123 (7) | 63.0± 11.0 | 776 (47.4) | Ach | >90% spasm & ischemic ECG | Myocardial lactate production |
| Suda (2019) | 187 | 128 (68) | 22 (12) | 63.2±12.3 | 74 (39.5) | Ach | >90% spasm, symptoms & ischemic ECG | Symptoms & ischemic ECG |
| Sueda (2015) | 416 | 72 (17.3) | NR | 64.4±10.8 | 193 (46.3) | Ach | >99% spasm, without additional criteria | NA |
| Sun (2005) | 131 | 69 (53) | 51 (39) | 59.8 | 69 (52.7) | Ach | >75% spasm, without additional criteria | 2 out of 3 (symptoms, ischemic ECG or myocardial lactate production) |
| Yamanaga (2014) | 50 | 24 (48.0) | NR | 61.3 | 24 (48.0) | Ach | >90% spasm, symptoms & ischemic ECG | NA |

Ach, acetylcholine; ANOCA, angina with no obstructive coronary arteries; Ergo, ergonovine; ECG, electrocardiogram; IQR, inter quartile range; n, number; NA, not applicable; NR, not reported; RCT, randomized controlled trial; SD, standard deviation.

**Table S6. Cardiovascular risk factors in ANOCA patients with and without microvascular spasm**

|  | **ANOCA patients**  **with MS**  **(n=283) %(95% CI)** | **ANOCA patients**  **without MS (n=183) %(95% CI)** | **Mean difference/OR**  **(95% CI) &**  **P Value** |
| --- | --- | --- | --- |
| **Age (mean years)** | 60 (52-68) | 60 (57- 64) | -0.18 (-12.24,11,88), p=0.954 |
| **Women (%)** | 64%  (27%-90%) | 48 %  (20%-77%) | 0.53 (0.03,8.06), p=0.508 |
| **Hypertension (%)** | 55%  (27%-80%) | 55%  (29%-79%) | 1.00 (0.82, 1.21), p=0.948 |
| **Dyslipidaemia (%)** | 38%  (18%-63%) | 38%  (15%-68%) | 1.05 (0.63,1.73), p=0.798 |
| **DM (%)** | 16%  (13%-20%) | 15%  (8%-26%) | 0.91 (0.53,1.57), p=0.618 |
| **Smoking (%)** | 30%  (20%-43%) | 25%  (21%-30%) | 1.20 (0.49,2.94), p=0.568 |

Four studies examining cardiovascular risk factors between ANOCA patients with and without microvascular spasm were included in this random effects meta-analysis. ANOCA, angina with no obstructive coronary arteries; CI, confidence interval; DM, diabetes mellitus; MS, microvascular spasm; OR, odds-ratio.

**Table S7. Cardiovascular risk factors in epicardial and microvascular spasm patients**

|  | **Epicardial spasm**  **(n=416)** | **Microvascular spasm**  **(n=283)** | **Mean difference/OR**  **(95% CI) &**  **P Value** |
| --- | --- | --- | --- |
| **Age (mean years, 95% CI)** | 62 (59-66) | 60 (52-68) | 2.02 (-7.36, 11.4), p=0.097 |
| **Women n(%)** | 57% (35%-77%) | 64% (27%-90%) | 0.69 (0.07,6.45), p=0.630 |
| **Hypertension (%)** | 56% (35%-75%) | 55% (27%-80%) | 0.90 (0.39, 2.09), p=0.710 |
| **Dyslipidaemia (%)** | 52% (38%-67%) | 38% (18%-63%) | 1.75 (0,63, 4.85), p=0.101 |
| **DM (%)** | 22% (13%-34%) | 16% (13%-20%) | 1,27 (0,74, 2,16), p=0.578 |
| **Smoking (%)** | 44% (24%-66%) | 25% (21%-30%) | 2,19 (1,10, 4.34), p=0.036 |

Four studies examining cardiovascular risk factors between epicardial and microvascular spasm were included in this random effects meta-analysis. ANOCA, angina with no obstructive coronary arteries; CI, confidence interval; DM, diabetes mellitus; OR, odds ratio.

**Table S8. Cardiovascular risk factors in female and male ANOCA patients with epicardial spasm**

|  | **Women**  **(n=980)**  **% (95% CI)** | **Men**  **(n=1457)**  **% (95% CI)** | **Mean difference/OR**  **(95% CI) &**  **P Value** |
| --- | --- | --- | --- |
| **Hypertension (%)** | 47% (5%-94%) | 48% (16-81%) | 1.10 (0.29,4.16), p=0.779 |
| **Dyslipidaemia (%)** | 41% (8% -85%) | 34% (4%-87%) | 1.13 (0.46,2.79), p=0.611 |
| **DM (%)** | 17% (14%-22%) | 21% (13%-32%) | 0.88 (0.65, 1.19), p=0.208 |
| **Smoking (%)** | 11% (1%-64%) | 62% (3%-99%) | 0.08 (0.00, 7.96), p=0.140 |

Three studies examining cardiovascular risk factors between male and female epicardial spasm patients were included in this random effects meta-analysis.

ANOCA, angina with no obstructive coronary arteries; CI, confidence interval; DM, diabetes mellitus; OR, odds ratio.

**Appendix 6.**

**
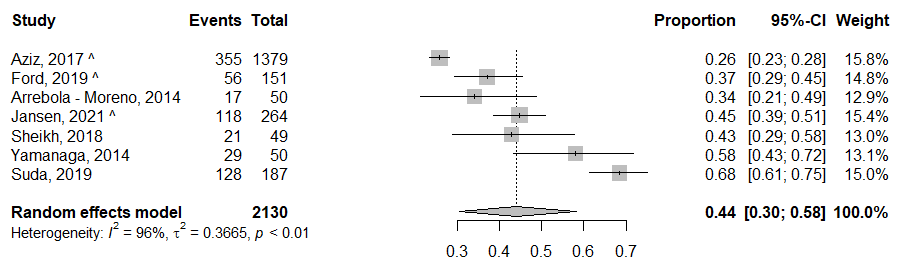
**

**Figure S1: Prevalence of epicardial spasm when studies used Acetylcholine as diagnostic agent and used the following diagnostic criteria; 1) epicardial vasoconstriction of 75% or 90%, 2) ischemic ECG changes, 3) symptoms.** Forest plot of published studies examining the prevalence of epicardial spasm with similar diagnostic criteria using random effects meta-analysis. Data presented as percentage (%) and 95% confidence intervals (CI; %).

**
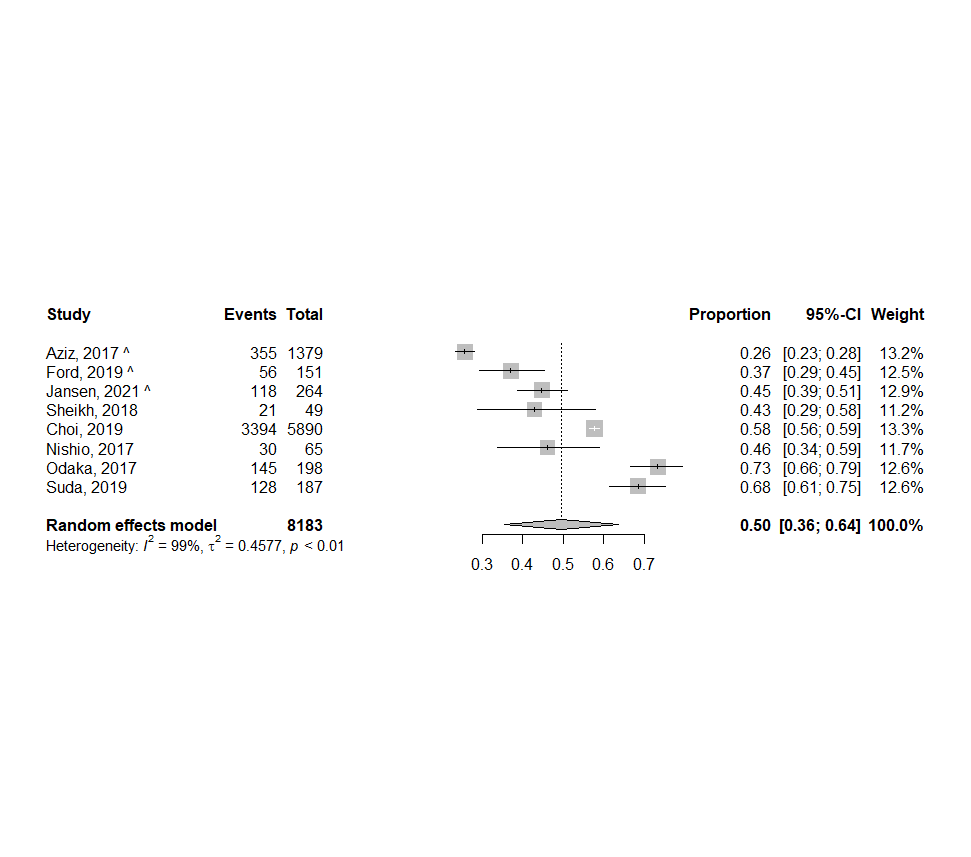
**

**Figure S2: Prevalence of epicardial spasm when only studies from 2017 or later are included in the analysis.** Forest plot of published studies examining the prevalence of epicardial spasm with similar diagnostic criteria using random effects meta-analysis. Data presented as percentage (%) and 95% confidence intervals (CI; %).

**Figure S3: Publication bias: funnel plot epicardial spasm**

**
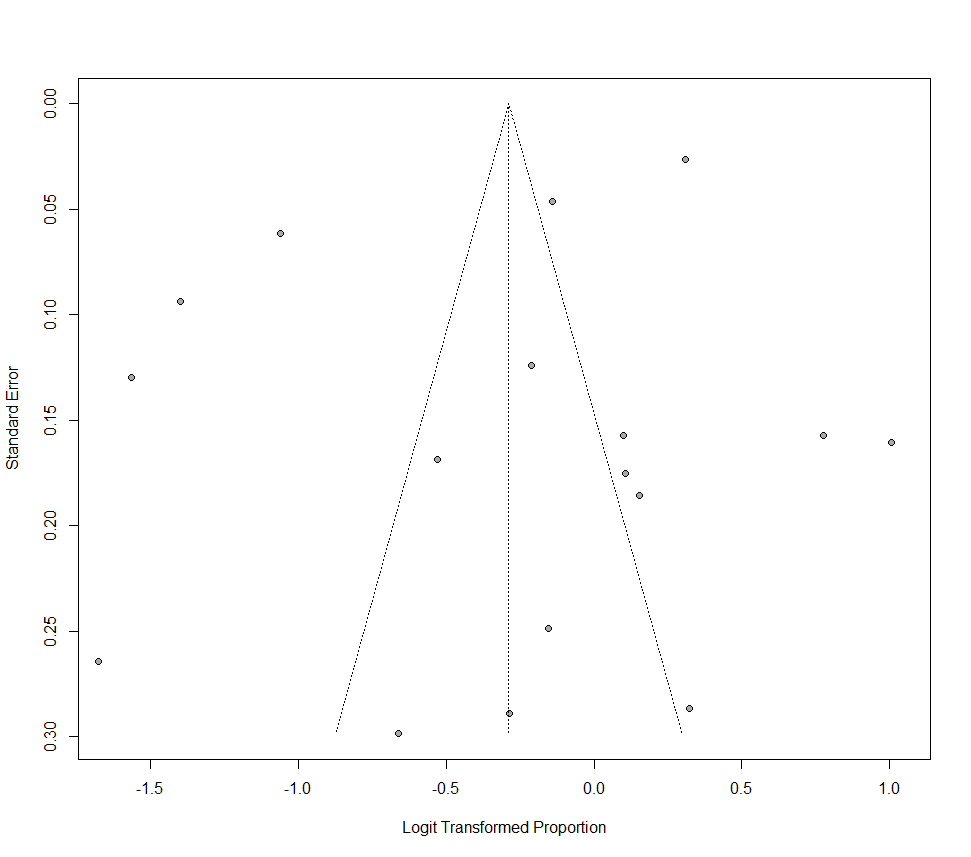
**

**Figure S4: Publication bias: funnel plot microvascular spasm**

**
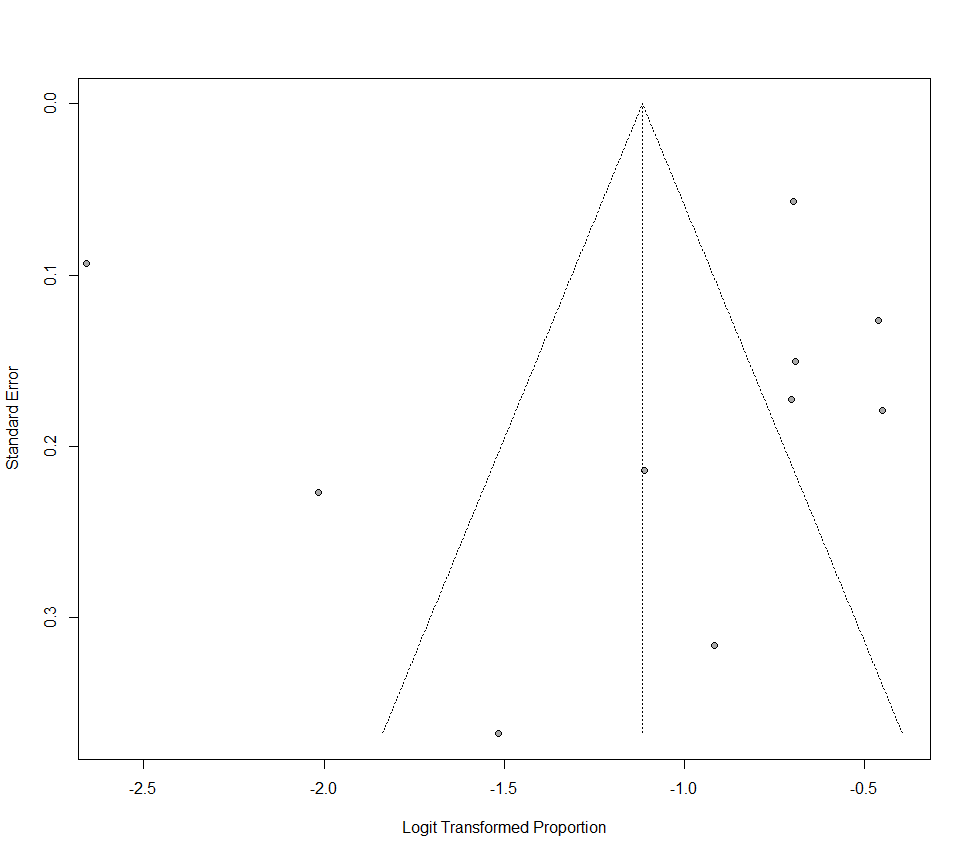
**

**
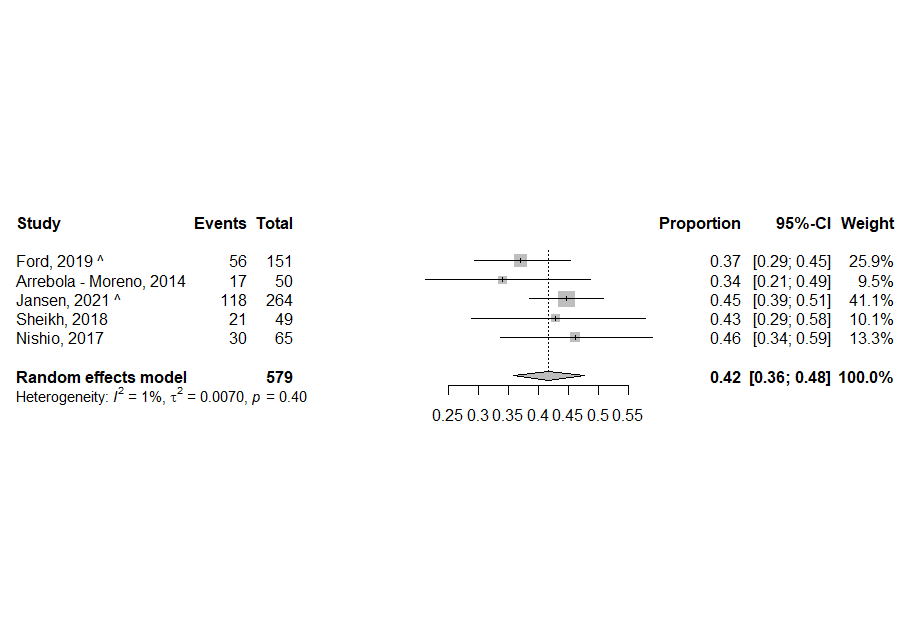
**

**Figure S5: Prevalence of epicardial spasm when only studies within the funnel plot are included in the analysis.** Forest plot of published studies examining the prevalence of epicardial spasm within the funnel plot using random effects meta-analysis. Data presented as percentage (%) and 95% confidence intervals (CI; %).

**
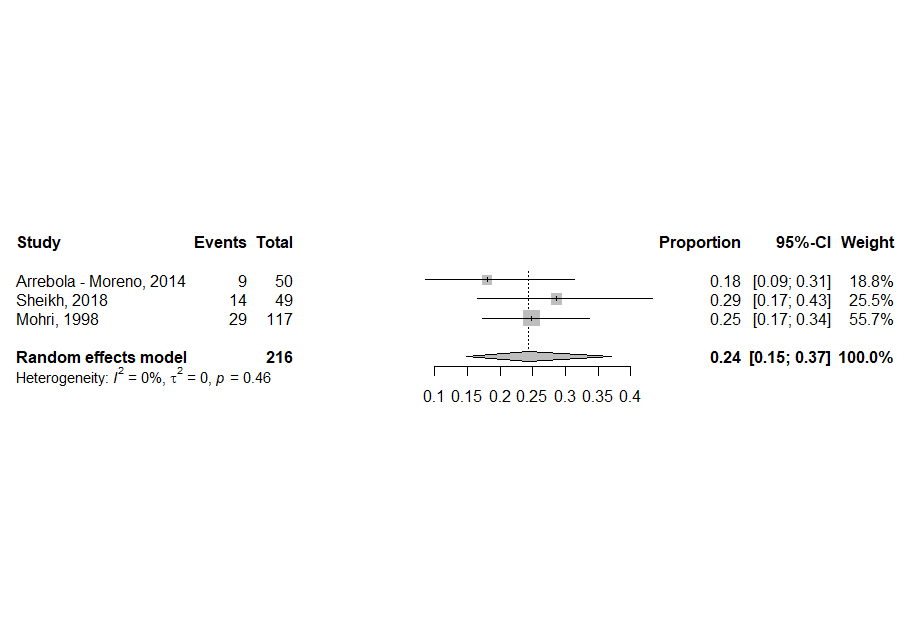
**

**Figure S6: Prevalence of microvascular spasm when only studies within the funnel plot are included in the analysis.** Forest plot of published studies examining the prevalence of microvascular spasm within the funnel plot using random effects meta-analysis. Data presented as percentage (%) and 95% confidence intervals (CI; %).
